# KIOM-4 Protects against Oxidative Stress-Induced Mitochondrial Damage in Pancreatic ****β****-cells via Its Antioxidant Effects

**DOI:** 10.1093/ecam/neq007

**Published:** 2011-06-08

**Authors:** Kyoung Ah Kang, Jin Sook Kim, Rui Zhang, Mei Jing Piao, Young Hee Maeng, Mi Young Kang, In Kyung Lee, Bum Joon Kim, Jin Won Hyun

**Affiliations:** ^1^School of Medicine, Jeju National University, Jeju-si 690-756, Republic of Korea; ^2^Diabetic Complication Research Center, Division of Traditional Korean Medicine Integrated Research, Korea Institute of Oriental Medicine, Daejeon, Republic of Korea; ^3^Department of Biomaterials, DNA Repair Center, Chosun University, Gwangju, Republic of Korea; ^4^Department of Microbiology and Caner Research Institute, Seoul National University College of Medicine, Seoul, Republic of Korea

## Abstract

The protective effect of KIOM-4, a mixture of plant extracts, was examined against streptozotocin (STZ)-induced mitochondrial oxidative stress in rat pancreatic **β**-cells (RINm5F). KIOM-4 scavenged superoxide and hydroxyl radicals generated by xanthine/xanthine oxidase and Fenton reaction (FeSO_4_/H_2_O_2_), respectively, in a cell-free chemical system. In addition, a marked increase in mitochondrial reactive oxygen species (ROS) was observed in STZ-induced diabetic cells; this increase was attenuated by KIOM-4 treatment. Mitochondrial manganese superoxide dismutase (Mn SOD) activity and protein expression were down-regulated by STZ treatment and up-regulated by KIOM-4 treatment. In addition, NF-E2 related factor 2 (Nrf2), a transcription factor for Mn SOD, was up-regulated by KIOM-4. KIOM-4 prevented STZ-induced mitochondrial lipid peroxidation, protein carbonyl and DNA modification. Moreover, KIOM-4 treatment restored the loss of mitochondrial membrane potential (Δ**ψ**) that was induced by STZ treatment, and inhibited the translocation of cytochrome c from the mitochondria to the cytosol. In addition, KIOM-4 treatment elevated the level of ATP, succinate dehydrogenase activity and insulin level, which were reduced by STZ treatment. These results suggest that KIOM-4 exhibits a protective effect through its antioxidant effect and the attenuation of mitochondrial dysfunction in STZ-induced diabetic cells.

## 1. Introduction

Mitochondria have gained importance in our understanding of diabetes because mitochondrial function is required for normal glucose-stimulated insulin release from pancreatic *β*-cells [1]. Mitochondria continuously generate superoxide radical as a byproduct of electron transport [2, 3]. The superoxide anion is quickly dismutated to hydrogen peroxide by mitochondrial manganese superoxide dismutase (Mn SOD) [4], and hydrogen peroxide is subsequently converted to water and oxygen by mitochondrial catalase and glutathione peroxidase [5]. Mitochondria not only produce reactive oxygen species (ROS) but are also the primary target of ROS attacks. Impaired mitochondrial function can lead to increased ROS generation and may increase oxidative stress if the antioxidant defense mechanisms of the cells are overwhelmed [6, 7]. Increased oxidative stress by mitochondrial dysfunction is considered a causal link between elevated glucose and the major biochemical pathways postulated to be involved in the pathogenesis of diabetes and diabetic complications [8, 9]. Streptozotocin (STZ) [*N*-(methyl nitro carbamoyl)-d-glucosamine] has been used to act as diabetogenic agent due to its ability to destruct pancreatic *β*-cells via the formation of ROS [10, 11]. It has been reported that production of mitochondrial ROS increased in STZ-treated rat, and mitochondrial lipid peroxidation was observed in pancreatic tissue, suggesting STZ-induced mitochondrial oxidative stress [12]. Thus, antioxidant therapy may be a promising therapeutic approach for controlling diabetes or diabetic complications.

KIOM-4 is a combination of extracts obtained from *Magnolia officinalis, Pueraria lobata, Glycyrrhiza uralensis* and *Euphorbia pekinensis. M. officinalis* exhibits antimutagenic, hepato-protective, neuro-protective, antiinflammatory and antimicrobial activities [13–18]. *P. lobata* has antimutagenic, antidiabetic and antioxidant effects [19–21]. *G. uralensis* has been documented as having detoxification, antioxidant, antiulcer, antiinflammatory, antiviral, antiatherogenic, anticarcinogenic effects and cytoprotective effect of hepatocyte against hepatotoxicity [22–24]. *E. pekinensis* has antiviral and cytotoxic activities [25, 26]. We recently demonstrated that KIOM-4 exhibits cytoprotective effects against STZ-induced oxidative stress damage in *β*-cells via the activation of catalase and heme oxygenase-1 [27, 28].

This study was undertaken to investigate the protective effect of KIOM-4 and its mechanism against STZ-induced oxidative mitochondrial damage in pancreatic *β*-cells.

## 2. Methods

### 2.1. Preparation of KIOM-4

The cortex of *M. officinalis*, and radixes of *P. lobata, G. uralensis* and *E. pekinensis* were collected from the Gamsuk province in China, and identified by Prof. J.H. Kim of the Division of Life Science, Daejeon University, Korea. All voucher specimens were deposited at the herbarium of the Department of Herbal Pharmaceutical Development, Korea Institute of Oriental Medicine (No. 1240, 2, 7 and 207, resp.). An equal amount of *Magnoliae* cortex, and radixes of *Puerariae*, *Glycyrrhizae* and *Euphoriae* was mixed, pulverized and extracted in 80% ethanol for one week at room temperature, concentrated using a rotary evaporator and lyophilized. The entire procedure was repeated four times. KIOM-4 was dissolved in dimethyl sulfoxide (DMSO), the final concentration of which did not exceed 0.1%.

### 2.2. Reagents

STZ was purchased from Calbiochem (San Diego, CA). Dihydrorhodamin 123 (DHR 123) and JC-1 (5,5′,6,6′-tetrachloro-1,1′,3,3′-tetraethyl-benzimidazolcarbocyanine iodide) were purchased from Molecular Probes (Eugene, OR). Cytochrome *c* (H-104), Nrf2 (C-20) antibodies were purchased from the Santa Cruz Biotechnology (Santa Cruz, CA). The Mn SOD polyclonal antibody was purchased from the Stressgen Corporation (Victoria, Canada). 5, 5-dimethyl-1-pyrroline-N-oxide (DMPO) and [3-(4,5-dimethylthiazol-2-yl)-2,5-diphenyltetrazolium] bromide (MTT) were purchased from the Sigma Chemical Company (St. Louis, MO).

### 2.3. Cell Culture and Treatments

RINm5F rat pancreatic *β*-cells were maintained at 37°C in an incubator with a humidified atmosphere of 5% CO_2_, and cultured in RPMI-1640 medium containing 10% heat-inactivated fetal calf serum, streptomycin (100 *μ*g ml^−1^) and penicillin (100 units ml^−1^). Cells were seeded on to a culture plate at a concentration of 1 × 10^5^ cells ml^−1^, and at 16 h after plating were treated with KIOM-4 at 50 *μ*g ml^−1^. After 1 h, 10 mM STZ was added to the plate.

### 2.4. Detection of Superoxide Radical

Superoxide radicals were produced by reaction of the xanthine/xanthine oxidase system and reacted with spin trap DMPO. The DMPO–*·*OOH adducts were detected using electron spin resonance (ESR) spectroscopy [29]. The ESR spectrum was recorded 2.5 min after mixing in a phosphate buffer solution (pH 7.4) with 6 M DMPO 20 *μ*l, xanthine oxidase (0.25 U ml^−1^) 20 *μ*l, xanthine (5 mM) 20 *μ*l and KIOM-4 (final 50 *μ*g ml^−1^) 20 *μ*l using JES-FA ESR spectrometer (JEOL, Tokyo, Japan).

### 2.5. Detection of Hydroxyl Radical

Hydroxyl radicals were generated by Fenton reaction. Hydroxyl radicals reacted with DMPO and the resultant DMPO–OH adducts were detected using an ESR spectrometer [29]. The ESR spectrum was recorded 2.5 min after mixing in a phosphate buffer solution (pH 7.4) with 0.2 ml of 0.3 M DMPO, 0.2 ml of 10 mM FeSO_4_, 0.2 ml of 10 mM H_2_O_2_ and KIOM-4 using ESR spectrometer.

### 2.6. Mitochondrial ROS Measurement

The RINm5F cells were seeded on to a 96-well plate at 2 × 10^4^ cells/well. At 16 h after plating, the cells were treated with KIOM-4 at 50 *μ*g ml^−1^, and 1 h later 10 mM STZ was added to the plate. The cells were incubated for an additional 30 min at 37°C. After addition of 20 *μ*M of DHR 123 solution for 10 min, the fluorescence was detected using a Perkin Elmer LS-5B spectrofluorometer and flow cytometer (Becton Dickinson, Mountain View, CA). For image analysis of the generation of mitochondrial ROS, the cells were seeded on a cover-slip loaded six-well plate at 2 × 10^5^ cells/well. At 16 h after plating, the cells were treated with KIOM-4, and 1 h later 10 mM STZ was added to the plate. After changing the media, 20 *μ*M of DHR 123 was added to each well and the plate was incubated for an additional 30 min at 37°C. After washing with PBS, the stained cells were mounted onto a microscope slide in mounting medium (DAKO, Carpinteria, CA). Images were collected using the Laser Scanning Microscope 5 PASCAL program (Carl Zeiss, Jena, Germany) on a confocal microscope.

### 2.7. Cellular Mitochondrial Fractionation

Mitochondrial fractions were isolated by differential centrifugation using the mitochondria isolation kit (Active-Motif, Carlsbad, CA).

### 2.8. Measurement of Mn SOD Activity

The RINm5F cells were seeded on to a culture dish at a concentration of 1 × 10^5^ cells ml^−1^, and at 16 h after plating were treated with KIOM-4 at 50 *μ*g ml^−1^. After 1 h, 10 mM STZ was added to the plate, which was incubated for a further 24 h. The harvested cells were suspended in 10 mM phosphate buffer (pH 7.5) and then lysed on ice by sonicating twice for 15 s. Triton X-100 (1%) was then added to the lysates and incubated for 10 min on ice. The lysates were clarified, by centrifugation at 5000 g for 30 min at 4°C, and the protein content of the supernatant was determined. Fifty micrograms of protein was added to 500 mM phosphate buffer (pH 10.2), 1 mM potassium cyanide (inhibitor of Cu Zn SOD) and 1 mM epinephrine. Epinephrine rapidly undergoes auto-oxidation at pH 10 to produce adrenochrome, a pink-colored product, which was assayed at 480 nm using a UV/VIS spectrophotometer in the kinetic mode. Mn SOD inhibits the auto-oxidation of epinephrine. The rate of inhibition was monitored at 480 nm and the amount of enzyme required to produce 50% inhibition was defined as one unit of enzyme activity, and the Mn SOD activity was expressed as units/mg protein [30].

### 2.9. Western Blot

Aliquots of the lysates (40 *μ*g of protein) were boiled for 5 min and electrophoresed on a 10% SDS-polyacrylamide gel. Blots in the gels were transferred onto nitrocellulose membranes (Bio-Rad, Hercules, CA). The nitrocellulose membrane was incubated with primary antibodies (1 : 1000) at 4°C overnight. Secondary antibody horseradish peroxidase conjugates (1 : 5000) (Pierce, Rockland, IL) were added, and incubated at room temperature for 1 h, and exposed to X-ray film. Protein bands were detected using an enhanced chemiluminescence western blotting detection kit (Amersham, Little Chalfont, Buckinghamshire, UK).

### 2.10. Lipid Peroxidation Assay

Lipid peroxidation was assayed by determination of 8-isoprostane levels [31] in the culture medium, by use of a commercial enzyme immunoassay kit (Cayman Chemical, Ann Arbor, MI) according to the manufacturer's instructions.

### 2.11. Protein Carbonyl Formation

The amount of protein carbonyl formation was determined using an Oxiselect protein carbonyl ELISA kit purchased from Cell Biolabs (San Diego, CA) according to the manufacturer's instructions.

### 2.12. 8-Hydroxyl-2′-deoxyguanosine Assay

The amount of 8-hydroxyl-2′-deoxyguanosine (8-OHdG) was determined using a Bioxytech 8-OHdG-ELISA Kit purchased from OXIS Health Products (Portland, OR) according to the manufacturer's instructions.

### 2.13. Mitochondrial Membrane Potential Analysis

Mitochondrial membrane potential (Δ*ψ*) was analyzed using JC-1, a lipophilic cationic fluorescence dye. Cells were harvested, and after changing the media, JC-1 was added to each well and incubated for an additional 30 min at 37°C. After washing with PBS, the stained cells were assayed using flow cytometer. For image analysis of mitochondrial Δ*ψ*, the stained cells were mounted onto microscope slide in mounting medium. Microscopic images were collected using the Laser Scanning Microscope 5 PASCAL program on confocal microscope [32].

### 2.14. Quantification of Cellular ATP Levels

The mitochondrial function was evaluated by measuring the cellular adenosine triphosphate (ATP) production in cells. The cells were harvested and washed twice with PBS. The harvested cells were then lysed on ice for 30 min in 200 *μ*l of lysis buffer [25 mM Tris (pH 7.8), 270 mM sucrose, 1 mM EDTA] by sonicating three times for 15 s and centrifuged at 4°C for 10 min at 16 000 g. Supernatants were collected from the lysates and ATP content was assayed using a luciferase/luciferin ATP determination kit (Molecular Probes, Eugene, OR) [33].

### 2.15. Mitochondrial Succinate Dehydrogenase Activity

To evaluate mitochondrial metabolic activity, mitochondrial succinate dehydrogenase activity was estimated by the MTT assay [34]. The cells were treated with KIOM-4 at 50 *μ*g ml^−1^. After 1 h, 10 mM of STZ, and the mixture was incubated for 24 h. Fifty microliters of the [3-(4,5-dimethylthiazol-2-yl)-2, 5-diphenyltetrazolium] bromide (MTT) stock solution (2 mg ml^−1^) was then added into each well to attain a total reaction volume of 200*μ*l. After incubating for 4 h, the plate was centrifuged at 800 *g* for 5 min and the supernatants were aspirated. The formazan crystals in each well were dissolved in 150 *μ*l of dimethylsulfoxide and read at A_540_ on a scanning multi-well spectrophotometer [35].

### 2.16. Measurements of Insulin Levels

The cells were treated with KIOM-4 at 50 *μ*g ml^−1^, and 1 h later 10 mM STZ was added to the plate, and the mixture was incubated for 24 h. The amount of insulin was determined by using an ELISA rat specific insulin enzyme immunoassay kit (Spi-Bio, Massy, France).

### 2.17. Statistical Analysis

All the measurements were made in triplicate and all values are represented as the mean ± standard error of the mean (SEM). Data were subjected to an analysis of the variance (ANOVA) using the Tukey test to analyze the difference. A *P*-value of <.05 was considered significant.

## 3. Results

### 3.1. Radical Scavenging Activity of KIOM-4 in a Cell-Free System

**Figure 1:**
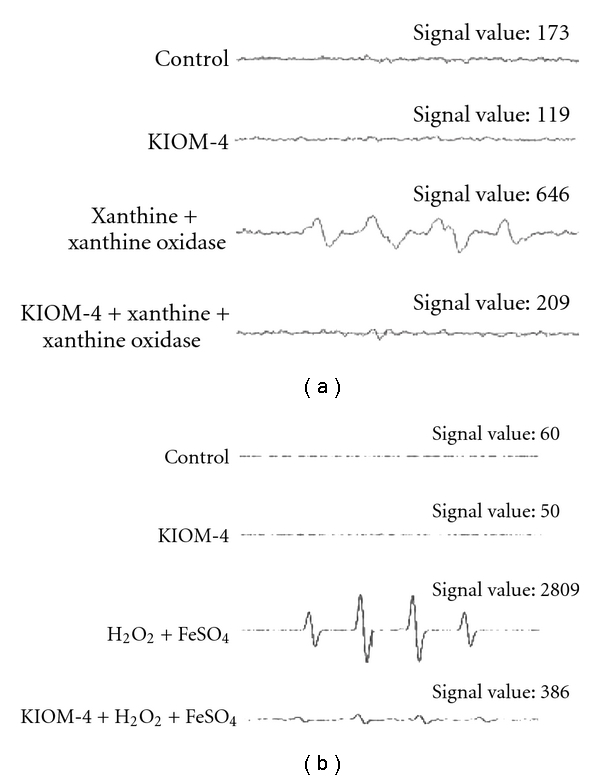
The effect of KIOM-4 on cell-free generation of superoxide and hydroxyl radicals. (a) Superoxide radicals were generated by xanthine/xanthine oxidase and reacted with DMPO; the resultant DMPO-OOH adducts were detected using an ESR spectrometer. (b) Hydroxyl radicals were generated by the Fenton reaction (H_2_O_2_/FeSO_4_) and reacted with DMPO; the resultant DMPO-OH adducts were detected using an ESR spectrometer.

Our previous result showed that the intracellular ROS scavenging activity of KIOM-4 was 48, 55 and 66% at concentrations of 10, 50 and 100 *μ*g ml^−1^, respectively [24]. And KIOM-4 at concentrations of 100 *μ*g ml^−1^ showed some cytotoxicity (data not shown). Therefore, we determined to choose 50 *μ*g ml^−1^ as optimal dose for further study. The radical scavenging effects of KIOM-4 on superoxide radicals and hydroxyl radicals were measured. Superoxide radicals produced by the xanthine/xanthine oxidase system and the hydroxyl radicals generated by the Fenton reaction (FeSO_4_ + H_2_O_2_) in a cell-free system were detected by ESR spectrometry. The ESR results revealed no clear signal in the control and the 50 *μ*g ml^−1^ of KIOM-4; however, the superoxide radical signal increased to 646 in the xanthine/xanthine oxidase system. KIOM-4 treatment decreased the superoxide radical signal to 209 (Figure 1(a)). In addition, the hydroxyl radical signal increased to 2809 in the FeSO_4_ + H_2_O_2_ system. KIOM-4 treatment decreased the hydroxyl radical signal to 386 (Figure 1(b)). 

### 3.2. Reduction of Mitochondrial ROS by KIOM-4 Treatment

**Figure 2:**
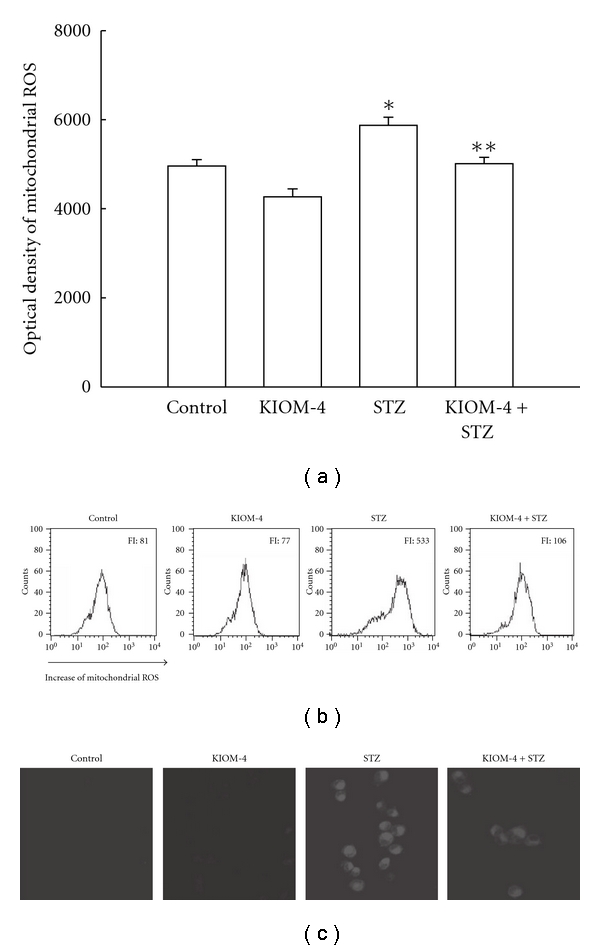
The effect of KIOM-4 on STZ-induced mitochondrial ROS generation. The cells were treated with KIOM-4 at 50 *μ*g ml^−1^. After 1 h, 10 mM of STZ was added to the plate. After an additional 30 min, the mitochondrial ROS were detected by spectrofluorometry (a) and flow cytometry (b) after DHR 123 treatment. FI indicates the fluorescence intensity of DHR 123. (c) The representative confocal images illustrate the increase in red fluorescence intensity of DHR 123 produced by ROS in STZ-treated cells s compared with that in control and the lowered fluorescence intensity in STZ-treated cells with KIOM-4 (original magnification ×400). The measurements were made in triplicate and the values were expressed as means ± SEM. Asterisk represents significantly different from control cells (*P* < .05) and double asterisk represent significantly different from STZ-treated cells (*P* < .05).

The fluorescence dye DHR 123 was used to detect mitochondrial ROS in cells after STZ treatment. The fluorescence spectrometric data revealed that STZ treatment increased the level of mitochondrial ROS compared with control. However, treatment with KIOM-4 at 50 *μ*g ml^−1^ attenuated the STZ-induced ROS increase (Figure 2(a)). In addition, flow cytometry revealed a fluorescence intensity of 106 for ROS in STZ-treated cells with KIOM-4 at 50 *μ*g ml^−1^, compared with a fluorescence intensity of 533 in STZ-treated cells (Figure 2(b)). Confocal microscopy revealed that KIOM-4 reduced the red fluorescence intensity of STZ-induced mitochondrial ROS (Figure 2(c)). These data suggest that KIOM-4 had mitochondrial ROS scavenging properties. 

### 3.3. Induction of Mn SOD and Its Transcription Factor by KIOM-4 Treatment

**Figure 3:**
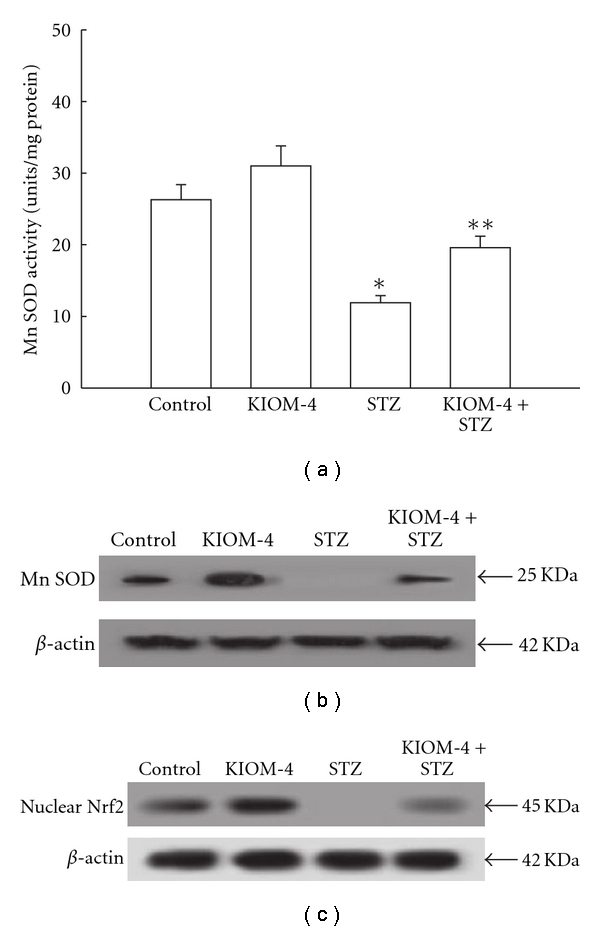
The effect of KIOM-4 on Mn SOD and its transcription factor. (a) The enzyme activities are expressed as average enzyme unit per mg protein ± SEM. Western blot analysis was performed using anti-Mn SOD (b) and Nrf2 (c) antibody. Asterisk represent significantly different from control cells (*P* < .05) and double asterisk represent significantly different from STZ-treated cells (*P* < .05).

Mn SOD acts as a first defense system to protect mitochondria and other cellular components as it scavenges superoxide anion in the mitochondrial matrix [36]. As shown in Figure 3(a), Mn SOD activity was 30 U mg^−1^ protein with 50 *μ*g ml^−1^ of KIOM-4, compared with 26 U mg^−1^ protein in the control. STZ treatment decreased the SOD activity to 12 U mg^−1^ protein; however, treatment with KIOM-4 increased this activity to 20 U mg^−1^ protein. In addition, western blot data revealed that STZ treatment decreased the expression of Mn SOD compared with control. KIOM-4 treatment at 50 *μ*g ml^−1^ increased the Mn SOD level attenuated by STZ treatment (Figure 3(b)). Mn SOD has an antioxidant response element (ARE) sequence in its promoter region. Nrf2 is an important transcription factor that regulates ARE-driven Mn SOD expression. Nuclear Nrf2 expression was decreased by STZ treatment; however, KIOM-4 treatment cells increased the nuclear Nrf2 expression (Figure 3(c)). These data suggest that KIOM-4 exhibited induction of Mn SOD via activation of Nrf2. 

### 3.4. Protection of Damaged Mitochondrial Components by KIOM-4 Treatment

**Figure 4:**
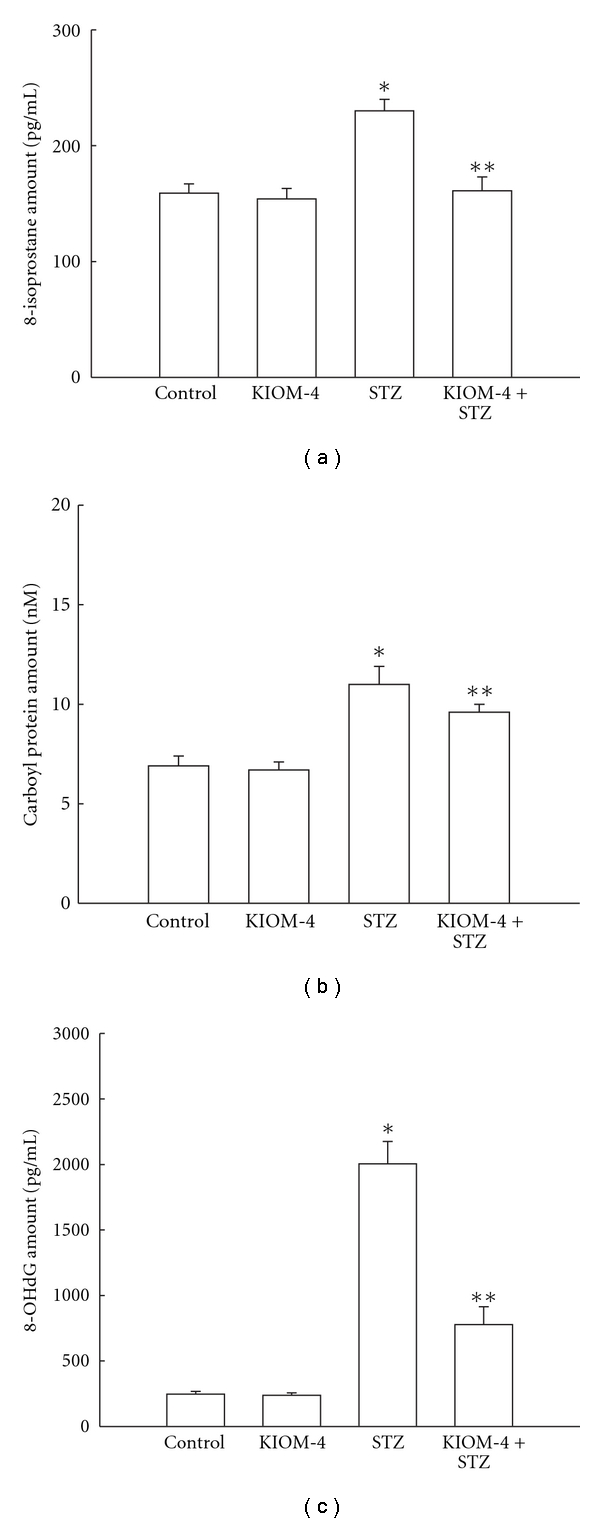
The effect of KIOM-4 on STZ-induced mitochondrial lipid, protein and DNA damage. (a) Lipid peroxidation was detected by measuring the amount of 8-isoprostane. (b) Protein oxidation was assayed by measuring carbonyl formation. (c) DNA damage was detected measuring the amount of 8-OHdG. Asterisk represent significantly different from control cells (*P* < .05) and double asterisk represent significantly different from STZ-treated cells (*P* < .05).

The level of 8-isoprostan, a marker of lipid peroxidation, is increased to 230 pg ml^−1^ in cells exposed to STZ, compared with 159 pg ml^−1^ in control cells. KIOM-4, however, decreased this level to 161 pg ml^−1^ in STZ-treated cells (Figure 4(a)). The mitochondrial protein carbonyl content, which is marker of protein modification [37], increased significantly after STZ treatment, and KIOM-4 prevented the STZ-induced protein carbonyl formation (Figure 4(b)). STZ treatment increased the amount of 8-OHdG, which is marker of base modification in DNA [38], to 2006 pg ml^−1^ compared with 247 pg ml^−1^ in control cells, and KIOM-4 decreased 8-OHdG to 777 pg ml^−1^ in STZ-treated cells (Figure 4(c)). These data suggest that KIOM-4 provides protection against STZ-induced mitochondrial damages. 

### 3.5. Recovery of Disrupted Mitochondrial Δ*ψ* and Its Related Protein by KIOM-4 Treatment

**Figure 5:**
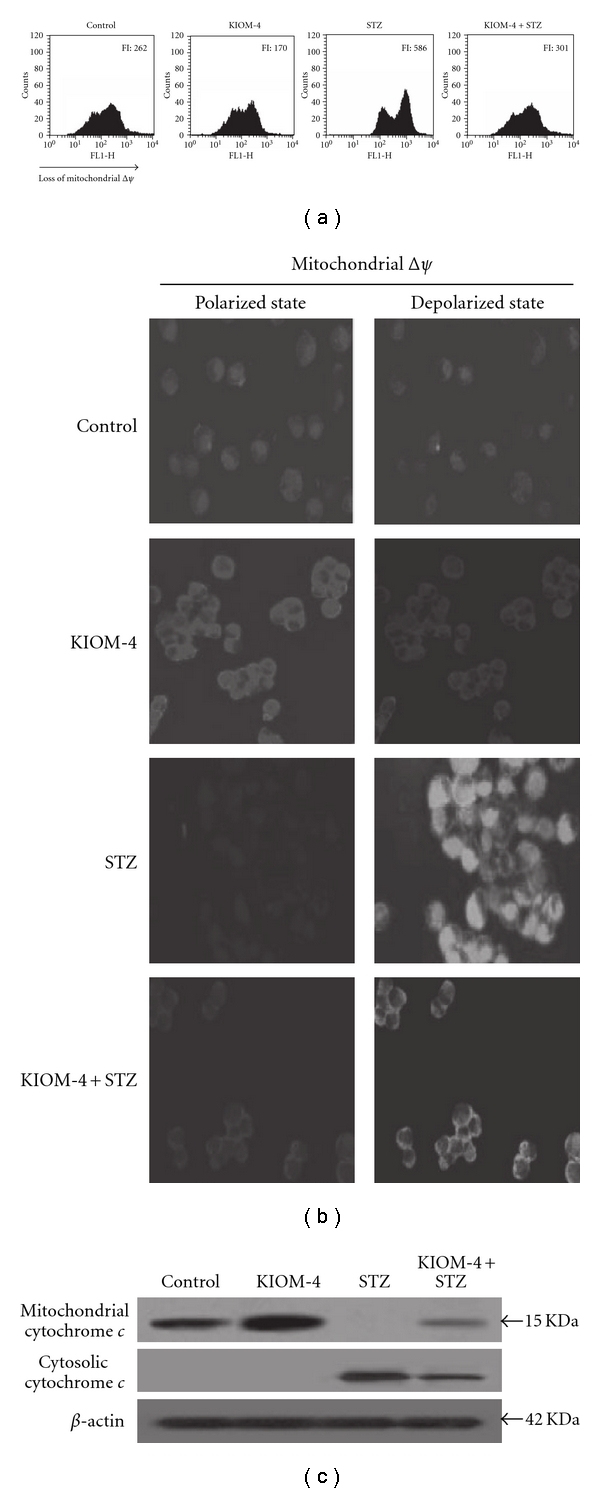
The effect of KIOM-4 on mitochondrial Δ*ψ* and its related proteins. The mitochondrial Δ*ψ* was analyzed using (a) flow cytometry and (b) confocal microscopy after staining cells with JC-1 dye. Western blot analysis was performed using anti-cytochrome c (c) antibody.

The mitochondrial Δ*ψ*, which is a marker of mitochondrial membrane integrity, was detected using flow cytometry and confocal microscopy after staining with the fluorescence dye JC-1. The flow cytometric data showed that the STZ treatment resulted in the loss of Δ*ψ*, as substantiated by an increase in fluorescence (FL-1) with JC-1. KIOM-4 treatment blocked the loss of Δ*ψ* in STZ-treated cells. The fluorescence intensity was 301 value in STZ-treated cells with 50 *μ*g ml^−1^ of KIOM-4, compared with a fluorescence intensity of 586 in STZ-treated cells (Figure 5(a)). In addition, the confocal microscopy data showed that control cells and cells treated with KIOM-4 only exhibited strong red fluorescence in the mitochondria after JC-1 staining, indicating that mitochondrial Δ*ψ* was in the polarized state (Figure 5(b), left panel). However, STZ treatment resulted in decreased red fluorescence in the mitochondria and increased green fluorescence, suggesting that STZ treatment disrupted the mitochondrial Δ*ψ* to a depolarized state. KIOM-4 treatment decreased the green fluorescence in STZ-treated cells (Figure 5(b), right panel), indicating that KIOM-4 inhibited the loss of Δ*ψ* in response to STZ treatment. The pore opening induces the loss of Δ*ψ*, which in turn induces the release of cytochrome c from the mitochondria and most commonly leads to apoptotic cell death [39]. KIOM-4 inhibited the STZ-induced release of cytochrome c from the mitochondria into the cytosol (Figure 5(c)). These results suggest that KIOM-4 protected against STZ-damage to mitochondrial Δ*ψ*. 

### 3.6. Enhancement of Decreased Intracellular ATP Level, Mitochondrial Enzymes, and Insulin Secretion by KIOM-4 Treatment

**Figure 6:**
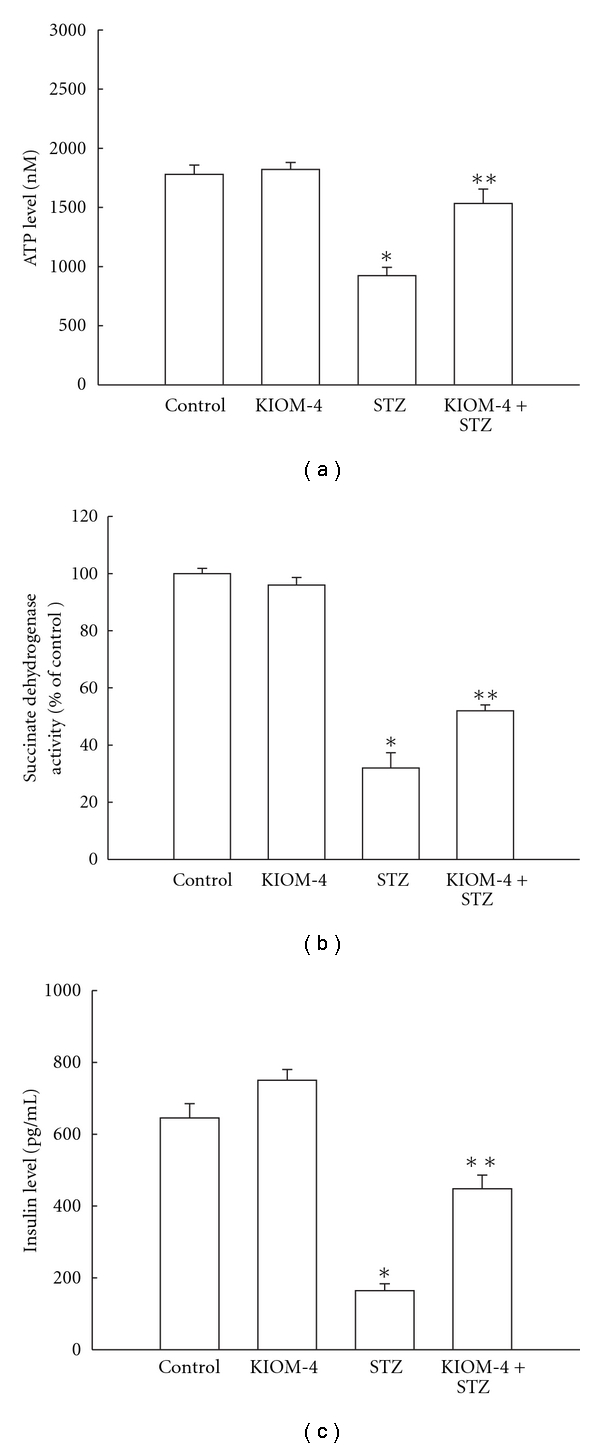
The effect of KIOM-4 on intracellular ATP level, mitochondrial enzyme, and insulin level. (a) ATP content was assayed using a luciferase/luciferin ATP determination kit. (b) Mitochondrial succinate dehydrogenase activity was estimated by the MTT assay. (c) The amount of insulin was determined by using an ELISA rat specific insulin enzyme immunoassay kit. Asterisk represent significantly different from control cells (*P* < .05) and double asterisk represent significantly different from STZ-treated cells (*P* < .05).

Mitochondrial injury is followed by the depletion of intracellular ATP and mitochondrial enzymes. As shown in Figure 6(a), STZ treatment reduced the ATP level compared with that in control cells; however, KIOM-4 treatment recovered the ATP level in STZ-treated cells. Mitochondrial succinate dehydrogenase activity was decreased in STZ-treated cells; however, KIOM-4 treatment of STZ-treated cells enhanced this activity (Figure 6(b)). Furthermore, STZ decreased the insulin level of RINm5F, which secretes insulin, however, KIOM-4 treatment of STZ-treated cells enhanced insulin secretion (Figure 6(c)). These results suggest that KIOM-4 attenuates mitochondrial dysfunction in STZ-induced diabetic cells. 

## 4. Discussion

Mitochondrial radical production and consequent oxidative damage contribute to the progressive and patho-physiological conditions of diabetes. It has been suggested that mitochondrial ROS induced by high glucose might cause the pathogenesis of diabetes mellitus and its complications through modification of various mitochondrial events [40]. Consequently, therapeutic strategies to decrease ROS production or to intercept these ROS should be explored [41]. Numerous studies have reported that antioxidant treatment, which targets oxidative stress, may help prevent or delay the development of diabetes and its complications [42, 43]. In this context, the possible anti-diabetic effects of KIOM-4 on the oxidative mitochondrial damage induced by STZ treatment in pancreatic *β*-cells were elucidated. In this study, KIOM-4 exhibited significant ROS radical scavenging activity against superoxide and hydroxyl radicals. Moreover, KIOM-4 attenuated the STZ-induced increase in mitochondrial ROS. Mn SOD is located in the mitochondrial matrix and is the primary SOD isoform that dismutates superoxide anions generated by the mitochondrial respiratory chain [44]. Pancreatic cells contain relatively low levels of antioxidant enzymes, making these cells more vulnerable to oxidative stress [42, 45]. A significant imbalance between ROS production and endogenous Mn SOD has been confirmed by the reduced activity and protein expression of Mn SOD in STZ-treated cells. In diabetes, decreased Mn SOD activity promotes the damage to cellular components such as lipid, protein and DNA [46]. Treatment with KIOM-4 restored the Mn SOD activity decreased by STZ. In addition, our previous study suggested that KIOM-4 exhibited cytoprotective effects against STZ-induced oxidative stress damage in *β*-cells via the activation of catalase and heme oxygenase-1 [27, 28]. Meanwhile, Nrf2 is able to activate the antioxidant-responsive element (ARE)-dependent gene expression in order to maintain cellular redox homeostasis [47]. Nrf2 is an important transcription factor that regulates ARE-driven expression of antioxidant genes, including Mn SOD. Nuclear Nrf2 expression was decreased by STZ treatment; however, KIOM-4 treatment recovered the nuclear Nrf2 expression. The components in the mitochondrial matrix are highly susceptible to an oxidative environment [48]. KIOM-4 prevented the STZ-induced mitochondrial lipid peroxidation, protein carbonyl and DNA modification. In general, mitochondrial DNA is more vulnerable to oxidative stress, and the subsequent damage is more extensive than that in nuclear DNA due to the lack of protective histones and low repair mechanisms [45, 46]. Elevated oxidative stress can increase the membrane permeability of the mitochondria by opening pores in the inner mitochondrial membrane, and can lead to the loss of Δ*ψ* [49]. The loss of mitochondrial Δ*ψ* can cause the release of cytochrome c and the activation of the apoptotic pathway [50]. Indeed, STZ treatment disrupted the mitochondrial Δ*ψ* and resulted in the release of mitochondrial cytochrome *c* to cytosol. However, KIOM-4 prevented the sequential mitochondrial damage process induced by STZ treatment. The elevated level of mitochondrial ROS generated by STZ acts as a powerful oxidant and causes damage to mitochondrial respiratory chain complexes. As mitochondria are the major producers of ATP, mitochondrial dysfunction also leads to reduced ATP levels [51]. Depletion of ATP content was observed in STZ-treated cells as a consequence of impaired mitochondrial respiratory chain activity. Succinate dehydrogenase is a mitochondrial TCA cycle enzyme and its activity is mainly regulated by ATP [52]. KIOM-4 treatment restored the ATP content and the succinate dehydrogenase activity, which were both reduced by STZ treatment. It has been reported that STZ treatment can increase mitochondrial ROS in rat pancreatic tissue, and the mitochondrial oxidative stress reduces insulin secretion by pancreatic *β*-cells [12, 53]. KIOM-4 significantly increased insulin content decreased by STZ treatment.

**Figure 7:**
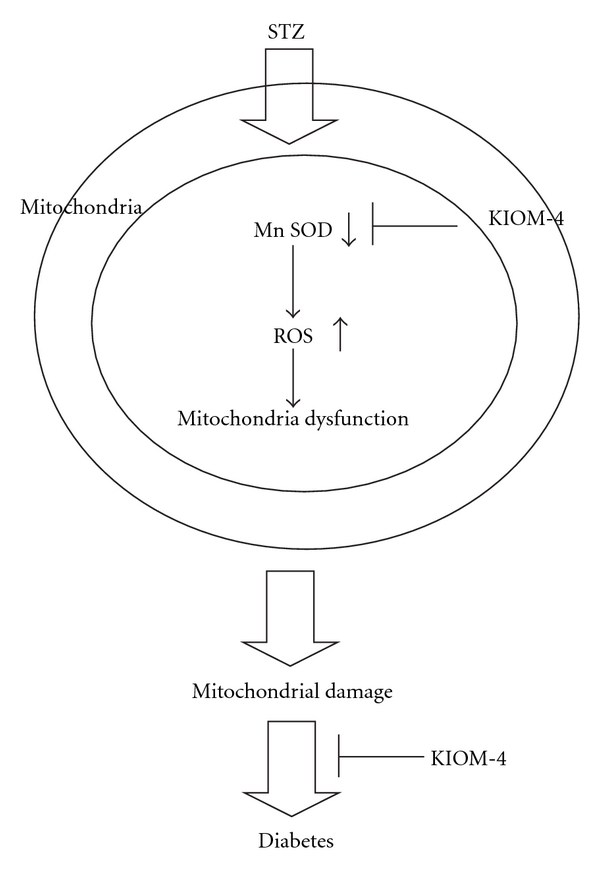
A proposed pathway for protective effect of KIOM-4 against STZ-induced mitochondrial oxidative stress in pancreatic *β*-cells via its antioxidant effects.

Hence, these results suggest that KIOM-4 protects against ROS-mediated mitochondrial dysfunction in diabetic pancreatic *β*-cells by scavenging ROS and inducing mitochondrial antioxidant enzymes (Figure 7).

## Funding

Korea Institute of Oriental Medicine (grant no. K09030).
